# Hemoglobin differences in twins are related to the time of cord clamping, not intertwin transfusion – a prospective cohort study

**DOI:** 10.1186/s12884-022-04942-2

**Published:** 2022-08-05

**Authors:** Katarzyna Kosińska-Kaczyńska, Jacek Witwicki, Aleksandra Saletra-Bielińska, Paweł Krajewski, Adam Krysiak, Robert Brawura-Biskupski-Samaha, Izabela Walasik, Magdalena Zgliczyńska, Ewa Malicka, Iwona Szymusik

**Affiliations:** 1 Department of Obstetrics, Perinatology and Neonatology, the Center of Postgraduate Medical Education, Ul. Cegłowska 80, 01-809 Warsaw, Poland; 2 2Nd Department of Obstetrics and Gynecology of, the Center of Postgraduate Medical Education, ul. Cegłowska 80, 01-809 Warsaw, Poland; 3 Department of Neonatology of the Center, Postgraduate Medical Education, Ul. Cegłowska 80, 01-809 Warsaw, Poland; 4 1St Department of Obstetrics and Gynecology, Medical University of Warsaw, pl. Starynkiewicza 1/3, 02-015 Warsaw, Poland; 5 Students’ Scientific Association at the 1st Department of Obstetrics and Gynecology, Medical University of Warsaw, pl. Starynkiewicza 1/3, 02-015 Warsaw, Poland

**Keywords:** Twin pregnancy, Delayed cord clamping, Early cord clamping, Neonatal outcomes, Twin-to-twin transfusion, Anemia, Polycythemia

## Abstract

**Background:**

Delayed cord clamping increases placental transfusion. In vaginal deliveries higher hemoglobin concentrations are found in the second-born twin. We hypothesized it is unrelated to intertwin transfusion but to the time of cord clamping. Methods: It was a prospective cohort study of 202 women delivering twins > 32 weeks of gestation. Monoamniotic pregnancy, antenatal intertwin transfusions, fetal demise or major abnormalities were excluded from the study. The time of cord clamping depended on the obstetrician’s decision. Hemoglobin, hematocrit, and reticulocyte count were measured at birth and during the second day of life.

**Results:**

At birth, hemoglobin and hematocrit levels were significantly higher in the first-born twins delivered with delayed than with early cord clamping. Higher hemoglobin and hematocrit levels were observed during the second day of life in all twins delivered with delayed cord clamping. The lowest levels were observed in twins delivered with early cord clamping. Infants delivered with delayed cord clamping were at a lower risk of respiratory disorders and NICU hospitalization.

**Conclusion:**

The observed differences in Hgb concentrations between the infants in a twin pregnancy are related to cord clamping time.

## Background

Delayed cord clamping (DCC) increases placental transfusion [1]. It allows the transfer of about 25–35 ml/kg of blood from the placenta to the newborn increasing neonatal volemia, hemoglobin (Hgb) concentration, and blood pressure in the first days of life [2]. A meta-analysis revealed that DCC decreased mortality in preterm infants [3–5]. DCC lowered the risk of intraventricular hemorrhage (IVH) and necrotizing enterocolitis (NEC) in preterm singletons [6, 7]. In a recent meta-analysis by Xodo et. Al. DCC was found to improve the long-term infants' neurological outcome [8]. In 2015, the International Liaison Committee on Resuscitation suggested performing DCC for over 30 s for preterm infants not requiring immediate resuscitation after birth, however it was a weak recommendation with very low-quality evidence [9]. However, data on DCC in twins are insufficient. Several studies on placental transfusion included newborns from multiple gestations, but the number of infants in each study was too low to draw conclusions [10, 11]. Few authors reported higher Hgb concentrations in the second-born twin after vaginal, but not cesarean delivery [12–14]. As the same relation was seen in monochorionic and dichorionic twins, we assumed that it was unrelated to placental anastomoses. It may be due to the fact that early cord clamping (ECC) is usually performed in both twins during cesarean section, while in vaginal birth ECC is performed in the first, and DCC in the second twin, and the observed Hgb differences are related to cord clamping time. Our study aimed to test this hypothesis.

## Methods

This prospective cohort study was conducted at two tertiary obstetric centers in Poland: 1st Department of Obstetrics and Gynecology, Medical University of Warsaw, and 2nd Department of Obstetrics and Gynecology, Center of Postgraduate Medical Education. The study was conducted according to the requirements of the Declaration of Helsinki for Medical Research involving Human Subject. Ethical approval was obtained from the Ethics Committee of the Medical University of Warsaw and the Ethics Committee of the Center of Postgraduate Medical Education. All participants gave their written informed consent to take part in the study.

The inclusion criteria were: patient’s age > 18 years, two live fetuses, delivery > 32 weeks of gestation. All cases of monoamniotic pregnancy, those diagnosed with twin-to-twin transfusion syndrome, twin anemia-polycythemia syndrome, fetal anemia, fetal demise, major fetal abnormalities, fetal genetic abnormalities, and antepartum or intrapartum hemorrhage were excluded from the study. If the first twin was born vaginally and the second via cesarean section, the pair was also excluded.

Postnatally, the cord clamping time of each twin depended on the obstetrician’s decision only and mostly resulted from their habits. There was no local policy on the time of cord clamping in twin delivery in any of the research centers. It was recorded by one of the researchers. The time of the first twin cord clamping was measured from delivery of the twin to the clamping of the cord. The time of the second twin was recorded also from delivery of the twin to the clamping of its cord. Before cord clamping, initial resuscitation steps had been performed and included providing warmth, drying the skin, clearing the airway, and stimulating the infant while waiting. If the infant did not respond to the resuscitation steps, the cord was clamped immediately, and the infant was handed over to the neonatology team. No cord milking was performed. All twins received standard postnatal care according to the local policy.

Cord clamping in both twins was followed by sampling 4 mL of umbilical blood from each cord. Hgb, Hct (hematocrit) and reticulocyte count (RC) were measured. RC helped differentiate between long-lasting and rapid intertwin transfusions. The intertwin RC ratio was calculated by dividing RC of one twin by the RC of the second twin. The second samples of 4 mL of neonatal venous blood were collected 24–48 h postnatally [13].

DCC was defined as cord clamping ≥ 30 s after birth and ECC < 30 s. The patients were divided into four groups: DCC performed in both infants (DCC + DCC group), ECC in both infants (ECC + ECC group), ECC in the first and DCC in the second twin (ECC + DCC group) and DCC performed in the first and ECC in the second twin (DCC + ECC group).

Gestational age was calculated on the first day of the last menstrual period, or the transfer day in assisted reproductive technique procedures and verified by the first-trimester crown-rump length. Small for gestational age (SGA) newborns were defined as ones weighing < 10th percentile for gestational age according to specific growth charts [15]. Polycythemia was defined as venous Hct levels above 65%. Intertwin Hgb differences were calculated and acute peripartum twin-to-twin transfusion was defined as Hgb difference > 8 g/dL according to previous studies [13, 14, 16].

Neonatal Hgb level at birth and on the second day of life was the primary outcome. Secondary outcomes included RC ratio and neonatal outcomes including polycythemia or anemia treated with blood transfusion, jaundice treated with phototherapy, IVH, NEC, respiratory disorders treated with continuous positive airway pressure (cPAP) or mechanical ventilation, Intensive Care Unit (NICU) admission. All infants were followed until discharge.

A power analysis was performed to assess sample size. In order to detect intertwin Hgb difference of 1 g/dL, a sample size of at least 160 twin pairs was required for the 80% power with error probability 0.05.

Variables were described as median, interquartile range (IQR) or number and per-centage and visualized with box and whisker plots. The Mann–Whitney test and the Fisher’s exact test were used for statistical analysis. *P*-values < 0.05 were considered significant. Data were analyzed using Statistica version 13.1.

## Results

**Fig. 1:**
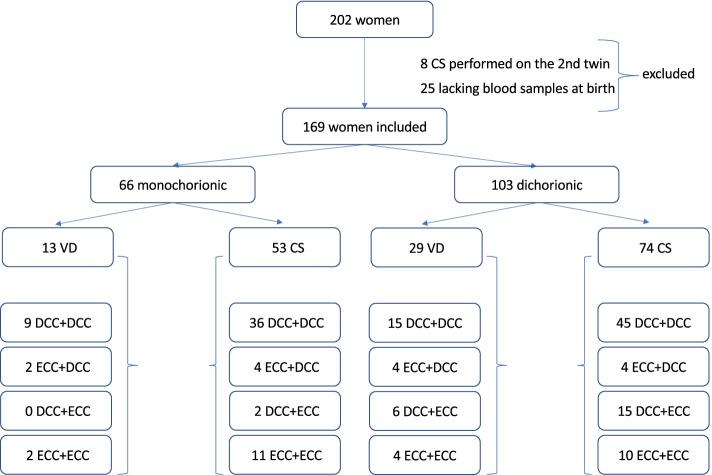
Flow diagram of the study group. CS – caesarean section; DCC + DCC – delayed cord clamping performed in both twins; DCC + ECC – delayed cord clamping in the first and early cord clamping in the second twin performed; ECC + DCC – early cord clamping in the first and delayed cord clamping in the second twin performed; ECC + ECC – early cord clamping performed in both twins

202 women were included in the study. 159 gave birth at the 1st Department of Obstetrics and Gynecology at the Medical University of Warsaw and 43 at the 2nd Department of Obstetrics and Gynecology at the Center of Postgraduate Medical Education between 2018 and 2021. 8 women were excluded due to cesarean section performed on the second twin only and 25 due to the lack of at least one umbilical blood sample. Finally, 169 women were included. All 338 twins had umbilical blood samples and 303 twins had venous blood samples collected. 105 women had both twins delivered with DCC, 12 DCC in the first and ECC in the second twin, 25 ECC in the first and DCC in the second twin and 27 ECC in both twins. 39.1% of twins were monochorionic. The basic characteristics of the study group are presented in Table 1 and the flow chart of the study group is visualized in Fig. 1.

**Table 1:** Basic characteristics of the study group

	Study group	DCC + DCC	DCC + ECC	ECC + DCC	ECC + ECC	p
**Mothers**						
	*N* = 169	*N* = 105	*N* = 12	*N* = 25	*N* = 27	
age (years)	30.7 (27.2–34.1)	29.8 (26.9–33.2)	30.3 (27.1–34.1)	30.1 (27.2–34)	31.1 (27.8–34.1)	0.9
primiparity	104 (61.5)	66 (62.8)	7 (58.3)	15 (60)	16 (59.3)	0.4
MC	66 (39.1)	45 (42.9)	4 (33.3)	4 (16)	13 (48.2)	0.01 ^*^
DC	103 (60.9)	60 (57.1)	8 (66.7)	21 (84)	14 (51.8)	0.01 ^*^
GH/PE	18 (10.7)	7 (6.7)	1 (8.3)	5 (20)	5 (18.5)	0.05
GDM	35 (20.7)	16 (15.2)	4 (33.3)	3 (12)	12 (44.4)	0.01 ^*^
ICP	15 (8.9)	10 (9.5)	1 (8.3)	2 (8)	2 (7.4)	0.8
PPROM	25 (14.8)	15 (14.3)	2 (16.7)	5 (20)	3 (11.1)	0.4
Gestational age at delivery (weeks)	36 (31–39)	36 (31–38)	35 (31–38)	36 (31–38)	36 (32–39)	0.8
PTD	93 (55)	51 (48.6)	10 (83.3)	14 (56)	18 (66.7)	0.03 ^**^
PTD < 34 wks	5 (3)	0	2 (16.7)	2 (8)	1 (3.7)	0.2
vaginal delivery	42 (24.9)	24 (22.8)	2 (16.7)	10 (40)	6 (22.2)	0.3
**Infants**						
	*N* = 338	*N* = 210	*N* = 24	*N* = 50	*N* = 54	
MC twins	132 (39.1)	90 (42.9)	8 (33.3)	8 (16)	13 (48.2)	0.01 ^*^
DC twins	206 (60.9)	120 (57.1)	16 (66.7)	42 (84)	28 (51.8)	0.01 ^*^
birthweight 1^st^ twin (g)	2575 (2030–2870)	2620 (2180–2910)	2350 (2030–2590)	2640 (2200–2890)	2450 (2080–2670)	0.1
birthweight 2^nd^ twin (g)	2510 (2000–2820)	2610 (2170–2930)	2200 (1980–2380)	2420 (2010–2740)	2360 (2060–2720)	0.1
SGA 1^st^ twin	23 (13.6)	12 (11.4)	2 (16.7)	4 (16)	5 (18.5)	0.3
SGA 2^nd^ twin	19 (11.2)	9 (8.6)	3 (25)	2 (8)	5 (18.5)	0.3
male twin	175 (51.8)	109 (51.9)	12 (50)	26 (52)	28 (51.9)	0.9
5^th^ minute Apgar < 8 of 1^st^ twin	8 (4.7)	6 (3.6)	0	0	2 (1.2)	0.2
5^th^ minute Apgar < 8 of 2^nd^ twin	14 (8.3)	6 (3.6)	2 (1.2)	1 (0.6)	1 (0.6)	0.1

Data are expressed as median (interquartile range) or number (%). p values are presented for the groups with the greatest difference. In small for gestational age infants and Apgar score the percentages are calculated for the number of the 1^st^ and 2^nd^ born twins

^*^ ECC + DCC vs ECC + ECC

^**^ DCC + DCC vs DCC + ECC

^***^ DCC + ECC vs ECC + ECC/DCC + ECC vs ECC + DCC

*DCC* + *DCC* delayed cord clamping performed in both twins, *DCC* + *ECC* delayed cord clamping in the first and early cord clamping in the second twin performed, *ECC* + *DCC* early cord clamping in the first and delayed cord clamping in the second twin performed, *ECC* + *ECC* early cord clamping performed in both twins, *MC* monochorionic gestation, *DC* dichorionic gestation, *GH* gestational hypertension, *PE* preeclampsia, *GDM* gestational diabetes mellitus, *ICP* intrahepatic cholestasis of pregnancy, *PPROM* preterm prelabor rupture of membranes, *PTD* preterm delivery, *MC twins* monochorionic twins, *DC twins* dichorionic twins, *wks* weeks of gestation

At birth the median Hgb level in the first-born twin was 17.9 g/dL (IQR 16.7–19.6) and the median Hct was 51% (IQR 47.2–55.9), while in the second-born twin the median Hgb was 18.3 g/dL (IQR 17.1–21.1; *p* = 0.3) and the median Hct was 52.4% (IQR 48.2–56.2; *p* = 0.4). During the second day of life in the first-born twin the median Hgb was 17.1 g/dL (IQR 16.4–18.5) and the median Hct was 48.5% (IQR 47.1–53.4), while in the second-born twin the median Hgb was 17.5 g/dL (IQR 16.7–19.1; *p* = 0.7) and the median Hct was 50.4% (IQR 44.1–55; *p* = 0.06).

**Fig. 2:**
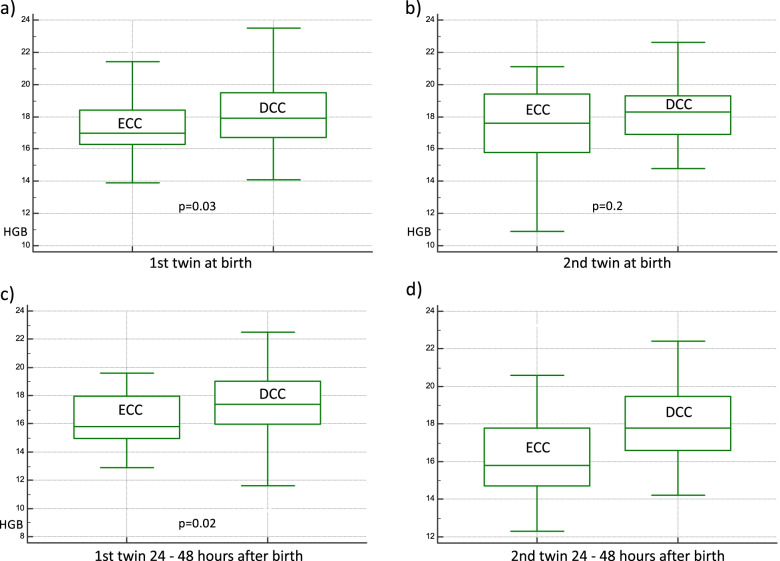
Hemoglobin levels in twins delivered with delayed and early cord clamping. **a** hemoglobin levels in the 1st twins at birth with early and delayed cord clamping; **b** hemoglobin levels in the 2nd twins at birth with early and delayed cord clamping; **c** hemoglobin levels in the 1st twins between 24 and 48 h after birth with early and delayed cord clamping; **d** hemoglobin levels in the 2nd twins between 24 and 48 h after birth with early and delayed cord clamping. ECC – early cord clamping; DCC – delayed cord clamping

247 twins were delivered with DCC and 91 with ECC. At birth Hgb and Hct levels were significantly higher in the first-born twins delivered with DCC than with ECC (Hgb: median 17.9 g/dL, IQR 16.7–19.5 vs 17 g/dL, IQR 16.3–18.4; *p* = 0.03; Hct: median 52.1%, IQR 48.2–56.1 vs 48.7%, IQR 47.1–53.6; *p* = 0.03). In the second-born twins significantly higher Hct was observed in DCC group (median 52.5%, IQR 49.4–55.8 vs 50.4%, IQR 44–54.9; *p* = 0.02). During the second day higher Hgb and Hct levels were observed in all twins delivered with DCC (first-born twin: Hgb 17.4 g/dL, IQR 17.1–18 in DCC group vs 15.8 g/dL, IQR 15–18; *p* = 0.002 in ECC group; second-born twin: 17.8 g/dL, IQR 17.3–18.2 in DCC group vs 15.8 g/dL, IQR 14.7–17.8; *p* =  < 0.001; first-born twin: Hct 50.2%, IQR 46.2–53.4 vs 46%, IQR 42.1–51.4; *p* = 0.003; second-born twin: 51.2%, IQR 47.5–55.5 vs 44.7%, IQR 41.7–50.9; *p* < 0.001). Figure 2 presents Hgb levels in twins delivered with DCC and ECC.

At birth polycythemia occurred in 7 monochorionic DCC newborns (2.8%) and in none delivered with ECC. However, during the second day of life no polycythemia was found in any group. In 3 cases polycythemia occurred in the first-born twin and they were all delivered with DCC. No anemia was found in the co-twins. In 2 cases polycythemia occurred in both twins and both were delivered with DCC.

Only 3 cases of Hgb difference > 8 g/dL were noted: 2 in monochorionic and 1 in a di-chorionic pregnancy. In 2 cases the first-born twin was delivered with DCC and the second-born with ECC, while in the third one both infants were delivered with DCC. No differences between monochorionic and dichorionic twins were observed (Table 2). No differences were observed in Hgb or Hct levels in twins delivered vaginally. In cesarean delivery the second-born twin had higher Hgb and Hct levels than the first one during the second day of life (Table 2).

**Table 2:** Hemoglobin, hematocrit, and reticulocyte count ration in twins in depending on chorionicity and mode of delivery

	Twin 1			Twin 2			
	HGB (g/dL) median (IQR)	HCT (%) median (IQR)	Reticulocyte count ratio median (IQR)	HGB (g/dL) median (IQR)	p	HCT (%) median (IQR)	p
**At birth**							
MC	18 (16.7–19.8)	51.5 (47.3–56.7)	0.9 (0.5–1.4)	18.2 (17.2–19.8)	0.9	52.1 (549.4–56.1)	0.8
DC	17.5 (16.5–18.9)	50.3 (47.1–55.2)	1 (0.6–1.4)	18.3 (17.6–18.5)	0.3	52.5 (48.1–55.4)	0.3
p	0.1	0.2	0.9	0.5		0.6	
**24–48 h after delivery**							
MC	17.1 (15.8–19)	48.8 (45.2–53.7)		17.8 (16.5–19.5)	0.2	51.3 (46.5–55.4)	0.2
DC	17 (15.6–18.7)	48.1 (44.8–53.4)		17.3 (15.9–19.3)	0.2	49.5 (45.3–53.7)	0.2
p	0.3	0.6		0.6		0.5	
**At birth**							
VD	18 (16.7–19.7)	52.8 (48.2–56.3)	0.9 (0.5–1.3)	17.5 (16.2–19)	0.2	49.7 (48.1–54.5)	0.2
CS	17.8 (16.5–19.1)	50.8 (46.7–55.6)	1 (0.5–1.4)	18.4 (16.9–19.8)	0.1	52.5 (48.8–56)	0.05
p	0.3	0.3	0.8	0.03		0.05	
**24–48 h after delivery**							
VD	16.8 (15.9–19.2)	48 (40–54.2)		17.2 (15.9–17.6)	0.8	48.2 (45.5–50.9)	0.7
CS	17.2 (15.6–18.8)	48.5 (44.9–53.4)		17.9 (15.9–19.5)	0.01	51.4 (46.2–55.6)	0.01
p	0.04	0.5		0.04		0.06	

*HGB* hemoglobin, *HCT* hematocrit, *IQR* interquartile range, *MC* monochorionic, *DC* dichorionic, *VD* vaginal delivery, *CS* cesarean section

**Fig. 3:**
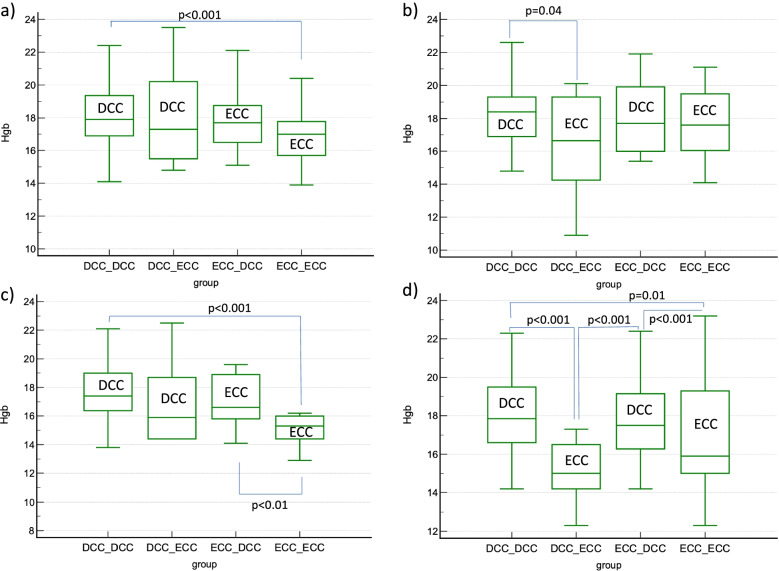
Hemoglobin levels in twins delivered with delayed and early cord clamping in all combinations. **a** hemoglobin levels in the 1st twins at birth with early and delayed cord clamping; **b** hemoglobin levels in the 2nd twins at birth with early and delayed cord clamping; **c** hemoglobin levels in the 1st twins between 24 and 48 h after birth with early and delayed cord clamping; **d** hemoglobin levels in the 2nd twins between 24 and 48 h after birth with early and delayed cord clamping. ECC – early cord clamping; DCC – delayed cord clamping

The results of blood samples of twins in relation to cord clamping time are presented in Table 3. Hgb levels are visualized in Fig. 3. The lowest Hgb and Hct levels were observed in ECC twins.

**Table 3:** Hemoglobin, hematocrit, and reticulocyte count ratio in twins in relation to the time of cord clamping

	Twin 1			Twin 2				
	HGB (g/dL) median (IQR)	HCT (%) median (IQR)	Reticulocyte count ratio median (IQR)	HGB (g/dL) median (IQR)	P (HGB 1^st^ twin vs 2^nd^ twin)	HCT (%) median (IQR)	P (HCT 1^st^ twin vs 2^nd^ twin)	HGB difference > 8 g/dL N (%)
**At birth**								
DCC + DCC *N* = 210	17.9 (16.9–19.4)	52.1 (48.6–56)	1 (0.5–1.4)	18.4 (16.9–19.3)	0.7	52.5 (49.4–55.7)	0.6	1
DCC + ECC *N* = 24	17.3 (15.5–20.2)	49.2 (43.6–56.3)	0.9 (0.4–1.4)	16.6 (14.3–19.3)	0.1	47.8 (41.6–54.9)	0.2	1
p^1^	0.6	0.4	0.9	0.04		0.04		1
ECC + DCC *N* = 50	17.7 (16.5–18.8)	50.7 (47.1–54.3)	1.1 (0.6–1.3)	17.7 (16–19.9)	0.7	52 (48.1–56.2)	0.5	0
p^2^	0.3	0.4	0.8	0.5		0.8		0.9
p^3^	0.8	0.5	0.2	0.2		0.04		0.9
ECC + ECC *N* = 54	17 (15.7–17.8)	48.5 (44.5–50.7)	1 (0.6–1.4)	17.6 (16.1–19.5)	0.3	50.4 (46–55.1)	0.3	1
p^4^	< 0.001	< 0.001	0.9	0.4		0.1		1
p^5^	0.8	0.7	1	0.2		0.3		1
p^6^	0.2	0.1	0.9	0.8		0.3		0.9
**24–48 h after delivery**								
DCC + DCC	17.4 (16.4–19)	50.4 (46.9–53.6)		17.9 (16.6–19.5)	0.2	51.2 (47.9–55.5)	0.1	1
DCC + ECC	15.9 (14.4–18.7)	46.2 (41.8–52)		15 (14.2–16.5)	0.08	42.7 (41.3–46.9)	0.1	1
p^1^	0.1	0.1		< 0.001		< 0.001		1
ECC + DCC	16.6 (15.8–19.9)	47.4 (45.9–53.9)		17.5 (16.3–19.2)	0.3	48.4 (47–55.8)	0.2	0
p^2^	0.2	0.3		0.9		0.8		0.9
p^3^	0.3	0.5		< 0.001		< 0.001		0.9
ECC + ECC	15.3 (14.4–16)	44.7 (40.7–47)		15.9 (15–19.3)	0.06	45.2 (42.9–52.5)	0.1	1
p^4^	< 0.001	< 0.001		0.01		0.04		1
p^5^	0.3	0.5		0.2		0.08		1
p^6^	< 0.01	< 0.01		< 0.001		< 0.001		0.9

p^1^ – DCC + DCC vs DCC + ECC

p^2^ – DCC + DCC vs ECC + DCC

p^3^ – DCC + ECC vs ECC + DCC

p^4^ – DCC + DCC vs ECC + ECC

p^5^ – DCC + ECC vs ECC + ECC

p^6^ – ECC + DCC vs ECC + ECC

*IQR* interquartile range, *DCC* + *DCC* delayed cord clamping performed in both twins, *DCC* + *ECC* delayed cord clamping in the first and early cord clamping in the second twin performed, *ECC* + *DCC* early cord clamping in the first and delayed cord clamping in the second twin performed, *ECC* + *ECC* early cord clamping performed in both twins, *MC* monochorionic gestation, *DC* dichorionic gestation

Neonatal outcomes were compared in monochorionic and dichorionic twins delivered with DCC and ECC and presented in Table 4. As regards monochorionic group, the first-born twins delivered with ECC suffered from respiratory disorders treated with cPAP more often than those delivered with DCC. The second-born dichorionic twins had respiratory disorders requiring mechanical ventilation and were hospitalized in NICU significantly more often in ECC group.

**Table 4:** Neonatal outcome in twins

	1^st^ twin			2^nd^ twin		
	DCC *N* = 117	ECC *N* = 52	p	DCC *N* = 130	ECC *N* = 39	p
	N (%)			N (%)		
**Monochorionic**	***N* = 49**	***N* = 17**		***N* = 49**	***N* = 17**	
Blood transfusion	0	0	1	0	0	1
Phototherapy	14 (28.6)	1 (5.9)	0.09	12 (24.5)	2 (11.8)	0.3
IVH I/II	2 (4.1)	0	0.4	0	0	1
IVH III/IV	0	0	1	0	0	1
NEC	0	0	1	0	0	1
CPAP	11 (22.4)	9 (52.9)	0.02	13 (26.5)	4 (23.5)	1
Mechanical ventilation	2 (4.1)	0	1	0	0	1
NICU	6 (12.2)	8 (47.1)	0.6	10 (2)	4 (23.5)	0.7
**Dichorionic**	***N* = 68**	***N* = 35**		***N* = 81**	***N* = 22**	
Blood transfusion	2 (2.9)	0	0.5	2 (2.5)	0	1
Phototherapy	15 (22.1)	8 (22.9)	0.9	6 (7.4)	4 (18.2)	0.1
IVH I/II	4 (5.9)	0	0.3	0	0	1
IVH III/IV	2 (2.9	0	0.5	0	0	1
NEC	2 (2.9)	0	0.5	0	0	1
CPAP	15 (22.1)	4 (11.4)	0.1	15 (18.5)	8 (36.4)	0.08
Mechanical ventilation	2 (2.9)	0	0.5	0	2 (9.1)	0.04
NICU	13 (19.1)	6 (17.1)	1	11 (13.6)	8 (36.4)	0.01

*DCC* delayed cord clamping, *ECC* early cord clamping, *IVH I/II* intraventricular hemorrhage grade I or II, *IVH III/IV* intraventricular hemorrhage grade III or IV, *NEC* necrotizing enterocolitis, *NICU* Neonatal Intensive Care Unit hospitalization

## Discussion

To our knowledge, it is the first study investigating neonatal outcomes in a large group of twins delivered with DCC or ECC in relation to chorionicity, twin birth order and the mode of delivery. We found Hgb and Hct levels to be related to cord clamping time. All DCC twins had significantly higher Hct levels. At birth, no differences were found in infants delivered vaginally or through cesarean section independently of the birth order, while during the second day of life significantly higher Hgb and Hct levels were found in the second-born twins delivered via cesarean section. No differences occurred between monochorionic and dichorionic twins. Furthermore, no differences in RC ratios were observed. Both monochorionic and dichorionic twin pairs were noted among ones with a large Hgb difference. We found DCC twins to be at a lower risk of respiratory disorders and NICU hospitalization.

Only one randomized controlled study of ECC versus DCC in twins was conducted and published by Ruangkit et al. 47 women were randomized into ECC or DCC during delivery. The researchers analyzed Hct levels on admission and at 8 weeks of age and found no differences between the groups. No cases of inter-twin Hgb difference > 8 g/dL occurred. The researchers concluded that DCC did not improve placental transfusion or increase systemic blood flow in multiple-birth infants [17]. However, the trial has several limitations, which encouraged us to perform the present study. The mean gestational age at delivery was 33 weeks, meaning most twins were delivered prematurely. The median gestational age was 36 weeks in our study. 55% of twins were delivered prematurely, but only 3% before 34 gestational weeks. Ruangkit et al. reported that 96% of ECC twins and 94% of DCC ones were born through cesarean section [17]. Therefore, the study group does not represent the population of twin deliveries. In our study every fourth twin pair was born vaginally.

There were 3 cases of Hgb difference > 8 g/dL in our study, one in dichorionic pregnancy. Hgb difference > 8 g/dL would be accounted by peripartum intertwin transfusion according to previous studies. If the observed differences resulted from twin-to-twin transfusion, they would be observed only in the monochorionic pregnancies due to the presence of anastomoses in monochorionic placenta. However, in our study they were observed in both monochorionic and dichorionic gestation. As anastomoses are extremely rare in dichorionic gestation we assumed that inter-twin Hgb difference resulted from differences in cord clamping time instead of perinatal twin-to-twin transfusion. Lopriore et al. analyzed the occurrence of inter-twin Hgb differences > 5 g/dL and found it in both monochorionic (2%) and dichorionic (8%) gestations. As regards twin pairs with a large Hgb difference, higher Hgb was found in both larger and smaller infants [12]. Verbeek et al. analyzed only monochorionic gestations and found Hgb difference > 8 g/dL in 5.2% of pregnancies, but only in vaginal deliveries. The second-born twins always had higher Hgb levels in those twin pairs [13]. As cord clamping time was not recorded in the above studies, the observed differences might be due to DCC and ECC.

The results of previous research vary. No differences in Hgb between DCC and ECC twins were found at birth in the only randomized trial. However, initial Hct levels may not reflect the actual blood shifts, as after acute blood loss they remain stable due to vasoconstriction. The second measurement after 8 weeks was most probably affected by previously performed blood transfusions or iron treatment [17]. Lopriore et al. found significantly higher Hgb levels in the second-born monochorionic twins [12]. Verbeek et al. analyzed monochorionic twins and found significantly higher Hgb levels in the second-born twins delivered vaginally [13]. They included both monochorionic and dichorionic twins in another study and found significantly higher Hgb levels in the second-born monochorionic and dichorionic twins when delivered vaginally, but no through cesarean section [14]. None of those studies reported cord clamping time. Obstetricians might tend to clamp the cord of the first-born twin more quickly in monochorionic gestations or vaginal delivery to focus on the delivery of the second twin. After the second twin was delivered its cord might be clamped later.

Several hypotheses explain the observed Hgb differences. The first two assume unbalanced inter-twin transfusion during delivery or placental-fetal transfusion towards the second-born twin due to the shared placenta. Our study and one by Lopriore et al. revealed marked Hgb differences in both monochorionic and dichorionic twins [12]. This hypothesis is unreal, as inter-twin transfusion is impossible without anastomoses. The third hypothesis suggests that uterine contractions and changes in fetal position during delivery may lead to inter-twin blood pressure differences and induce inter-twin transfusion during delivery. Again, the existence of anastomoses is necessary. Furthermore, our study revealed differences in Hgb and Hct levels during the second day of life in infants delivered through cesarean section and not vaginally. The fourth hypothesis assumes that inter-twin Hgb differences are correlated with cord clamping time. Our results confirm this hypothesis.

The strengths of our study include its prospective and two-center nature. A large study group, whose number was confirmed with power analysis, ensures that results are relevant and allow meaningful conclusions to be drawn. Two measurements of Hgb and Hct levels at an interval of time and RC were essential to the analysis of possible perinatal blood shifts. The inclusion of monochorionic and dichorionic twins enabled us to verify the hypothesis of inter-twin blood transfusion during delivery. However, the study has several limitations. Neither the participants, nor the obstetric stuff were blinded for the intervention, as it was impossible to perform. Cord clamping time depended only on the obstetrician’s decision and was not randomized. No inter-twin delivery interval was recorded. Therefore, it is possible impact on Hgb levels could not be analyzed. However, Bhide et al. demonstrated no correlation between inter-twin delivery interval and Hgb concentrations [18].

In 2018, Italian Recommendations for Placental Transfusion Strategies were published. They included guidelines on twin pregnancy. DCC was not recommended in mono-chorionic twins, because of the risk of acute inter-twin transfusion, while in dichorionic twins it was suggested [1]. Our study brings novel insight into the problem. Its unique results help verify the hypotheses explaining the observed differences in Hgb concentrations between twins and confirm the hypothesis of the impact of cord clamping time. We have proved that that DCC for both twins if feasible and safe to do, even in monochorionic gestation. However, prospective randomized trials on cord clamping time in twin gestations are needed to revise the guidelines.

## Conclusions

The observed differences in Hgb concentrations between the twins delivered > 32 weeks of gestation with no diagnosed antenatal intertwin transfusion are related to cord clamping time. Delayed cord clamping may improve neonatal outcomes in twins.

## Data Availability

The datasets used and/or analysed during the current study are available from the corresponding author on reasonable request.

## Abbreviations

DCC: Delayed cord clamping
Hgb: Hemoglobin
IVH: Intraventricular hemorrhage
NEC: Necrotizing enterocolitis
ECC: Early cord clamping
Hct: Hematocrit
RC: Reticulocyte count
SGA: Small for gestational age
cPAP: Continuous positive airway pressure
NICU: Neonatal Intensive Care Unit
IQR: Interquartile range
